# Comparison of epidural ropivacaine and ropivacaine clonidine combination for elective cesarean sections

**DOI:** 10.4103/1658-354X.65119

**Published:** 2010

**Authors:** Sukhminder Jit Singh Bajwa, Sukhwinder Kaur Bajwa, Jasbir Kaur

**Affiliations:** *Gian Sagar Medical College and Hospital, Banur, Patiala, Punjab, India*

**Keywords:** *Clonidine*, *cesarean section*, *epidural anesthesia*, *ropivacaine*

## Abstract

**Background and Aim:**

Neuraxial adjuvants augment the action of local anesthetics. The aim is to determine the qualitative and quantitative aspects of epidural block of ropivacaine 0.75% versus ropivacaine 0.75% with clonidine for elective cesarean section.

**Settings and Design:**

A randomized double-blind study was conducted among 51 healthy parturients, scheduled for elective cesarean section, at Gian Sagar Medical College and Hospital, Banur, Punjab, India.

**Materials and Methods:**

Epidural block was administered with 20 ml of ropivacaine 0.75% (group R) and ropivacaine 0.75% and clonidine 75 *µ*g (group RC) and anesthetic level was achieved minimum until T6–T7 dermatome. Onset time of analgesia, sensory and motor block levels, maternal heart rate and blood pressure, neonatal Apgar scores, postoperative analgesic dose and adverse events were recorded.

**Results:**

Fifty one patients were enrolled in this study and were subjected to statistical analysis. Groups were comparable with regard to demographic data, neonatal Apgar scores and incidences of side effects except for the higher incidence of dry mouth in patients of RC group. Onset of analgesia was much shorter in RC group along with prolonged duration of analgesia. The incidence of bradycardia and hypotension was more in RC group as compared to R group which was statistically significant. The dose requirement for postoperative pain relief was significantly lesser in RC group.

**Conclusions:**

The addition of 75 *µ*g clonidine to isobaric epidural ropivacaine results in longer, complete and effective analgesia with similar block properties and helped to reduce the effective dose of ropivacaine when compared with plain ropivacaine for cesarean delivery.

## INTRODUCTION

Bupivacaine has been studied extensively in labor analgesia and anesthesia. In recent years, ropivacaine has been increasingly replacing bupivacaine for the said purpose because of its similar analgesic properties, lesser motor blockade and decreased propensity of cardiotoxicity.[1] Though a slightly larger dose of ropivacaine is required as compared to bupivacaine to achieve the analgesic and anesthetic effects, the addition of adjuvant can decrease the dose of ropivacaine required thereby eliminating quite a few side effects associated with larger doses of ropivacaine.[2]

Clonidine has been used as an adjuvant in regional anesthesia in various settings.[3,4] It is an α-2 adrenergic agonist that produces analgesia via a non-opioid mechanism. The combination of epidural clonidine with bupivacaine for labor analgesia has been extensively studied, and it has been shown to improve analgesia when added to epidural ropivacaine but there is hardly any study which has compared the effects of addition of epidural clonidine with those of ropivacaine for cesarean section. Similarly, the coadministration of clonidine and local anesthetic produced better analgesia than either drug alone.[5,6]

There are a few studies which have shown comparison of bupivacaine and ropivacaine but addition of clonidine as an adjuvant helps in sparing of doses of either drug, which consequently reduces the incidence of side effects associated with larger doses of these anesthetics.[7,8]

This study aims to determine the qualitative and quantitative aspects of epidural block, hemodynamic effects, effect on Apgar score and postoperative pain relief of ropivacaine 0.75% versus ropivacaine 0.75% with clonidine for elective cesarean section.

## MATERIALS AND METHODS

After the approval of ethics committee, written informed consent of the patients and their relatives were taken. Fifty one healthy pregnant females of American Society of Anaesthesiologist (ASA) physical status I and II, scheduled for elective cesarean section, were enrolled in this randomized double-blind study. All had full term (>36 and <40 gestation weeks) singleton fetus with an estimated fetal weight of >2300 g and were aged >20 years and less than 34 years with body weights in the range 55–90 kg and were of height >150 cm. Women requesting and consenting for epidural anesthesia were considered for inclusion in the study. The parturients with maternal cardiac disease, maternal hematological disease, bleeding or coagulation test abnormalities, fetal distress, known fetal anomalies, pregnancy-induced hypertension, multiple pregnancies, psychiatric diseases, gestational diabetes, history of drug abuse and allergy to local anesthetics of the amide type, were excluded from the study.

All the patients were administered premedication in the form of tablet ranitidine 150 mg a night before and in the morning 2 hours before surgery with a sip of water. In the operation theatre, a good IV access was secured and patients were preloaded with 750 mL of lactated ringer's solution. Monitoring gadgets were attached to monitor ECG, heart rate, Non-Invasive Blood Pressure (NIBP) and SpO_2_. Baseline hemodynamic parameters, respiratory rate, ECG and SpO_2_ were recorded. Women were randomly assigned in a double-blinded fashion to one of the two groups with the help of a computer-generated code whereby numbered envelopes were assigned to each patient. The syringes used in the study were prepared by an anesthesia technician who was not a part of the operation theatre team. Women were placed in the sitting position, and local anesthesia of skin and subcutaneous tissues was performed at lumbar level L3–4 with lignocaine HCl 2% 1–2 mL Thereafter, the epidural space was localized and confirmed with the loss of resistance to saline technique using an 18-gauge Touhy needle. An epidural catheter was then inserted into the epidural space in a cephalic direction and aspirated for detection of cerebrospinal fluid or blood. After the catheter was secured to skin surface, subjects were repositioned with left uterine displacement by keeping a wedge beneath the right half of lower back and a pillow was placed below the head and shoulders. Thereafter, 3 mL of 2% lignocaine HCl with 1 in 2 lakh adrenaline solution was administered as a test dose and any untoward effect was observed for. After 5–7 minutes of institution of the test dose, group R received epidural anesthesia with 20 mL of 0.75% ropivacaine, whereas group RC received 20 mL of 0.75% ropivacaine and clonidine 75 µg. Surgical procedures were initiated only after the anesthetic level was completely established minimum until T6–T7. The sensory level was checked and confirmed with pin-prick method bilaterally at 5, 10, 15, 20, 25 and 30 minutes. Motor block using a modified Bromage scale (0 = no block, 1 = inability to raise extended leg, 2 = inability to flex knee and 3 = inability to flex ankle and foot) was also recorded at the same intervals. The following variables were recorded: time to initial onset of analgesia, highest level of sensory analgesia, the time to complete motor block, time to two-segment regression of analgesic level from T6 dermatome, regression of analgesic level to L5 dermatome and time to complete recovery.

Maternal hemodynamic parameters, which included heart rate, ECG, NIBP (both systolic and diastolic), respiratory rate and SpO_2_, were monitored continuously. Initial bolus dose timing was assumed to be as baseline time. Recordings were made every 5 minutes until 30 minutes and at 10-minute intervals thereafter up to 60 minutes and then at 15-minute intervals for the next hour and finally at 30 minutes at the third hour. Hypotension (defined as systolic arterial pressure falling more than 20% mmHg) was treated with injection mephenteramine 3–6 mg in bolus doses and heart rate <55 beats/minute was treated with 0.3 mg of injection atropine. Intravenous fluids were given as per the body weight and operative loss requirement with no patient requiring blood transfusion. Patients were given supplementary O_2_ with the help of venturi mask. During the surgical procedure, adverse events like anxiety, nausea, vomiting, pruritis, shivering, maternal bradycardia or hypotension were recorded. Nausea and vomiting were treated with 4–6 mg of i.v. ondansetron.

During and at the end of surgery, the overall quality of anesthesia was judged by the anesthetist and the gynecologist on a numeric rating scale (NRS) from 1 (unsatisfactory) to 5 (excellent). Maternal vitals were recorded in the recovery room also at 1, 5, 10, 20 and 30 minute interval. The onset of pain was managed by top-up doses of 8 mL of 0.175% ropivacaine in R group, whereas 50 µg clonidine was added to the same volume of ropivacaine in RC group. Neonatal condition was evaluated by Apgar scores at 1, 5 and 10 minutes after delivery. An Apgar score of less than 7 was considered abnormal in this project.

The data were analyzed statistically. The variables (maternal hemodynamic parameters and block characteristics) in the two groups were compared using the chi-squared test and *P* value. Student's t-test was used to compare maternal hemodynamic parameters. For all the statistical analysis, the level of significance was *P* < 0.05. Neonatal Apgar scores were tested by multiple linear regression analysis.

## RESULTS

Fifty six patients were enrolled in this study and five cases were excluded [one patient was given general anesthesia two patients had patchy effect (pain) and in two cases epidural catheter had migrated into subarachnoid space] and the data of 51 of them were eligible and were processed for analysis: 27 in group RC and 24 in group R [Table 1].

**Table 1:** Comparison of demographic profile of the R and RC groups

	Ropivacaine (*n* = 24)	Ropivacaine + clonidine (*n* = 27)
Age (years)	23.90 ± 5.27	24.30 ± 7.10
Height (cm)	160.60 ± 5.50	159.10 ± 7.75
Weight (kg)	74.88 ± 10.46	72.06 ± 9.55
Body mass index	29.63 ± 4.62	28.08 ± 3.35
ASA (I/II)	21/3	23/4
Duration of surgery (minutes)	32.56 (28.34–48.40)	34.42 (27.28–46.58)

The two groups were comparable with regard to demographic data as shown. Duration of surgery was comparable in both the groups and did not show any significant variation [Table 2].

**Table 2:** Comparison of initial block characteristics

	R (*n* = 24)	RC (*n* = 27)	*P*
Onset time at T10–T11 (minutes)	11.36 ± 3.30	8.64 ± 2.56	<0.05
Sensory blockade level	T6–T7	T5–T6	
Time to sensory blockade at T6–T7	15.12 ± 4.36	12.26 ± 3.18	<0.05
Time to complete motor block (minutes)	21.70 ± 4.20	17.34 ± 4.48	<0.05

Onset of anesthesia was shorter in group RC as compared to group R. However, once sensory level was established at T6–T7 level, there was no noticeable difference in sensory anesthesia in either of the groups throughout the surgical procedure. The establishment of complete motor blockade was much earlier in the RC group which was again statistically significant (*P* < 0.05) [Table 3].

**Table 3:** Comparison of mean pulse rate at different time intervals in groups R and RC

Time interval (minutes)	Groups	Mean ± SD	Pulse rate	
			*t*	*P*
Baseline (1 minute)	R	80.24 ± 4.66	—	>0.05
	RC	82.16 ± 5.22		
5	R	82.14 ± 5.46	4.70	>0.05
	RC	84.16 ± 4.88		
10	R	86.36 ± 6.20	7.56	<0.05
	RC	84.26 ± 4.44		
15	R	86.44 ± 5.84	2.42	>0.05
	RC	86.48 ± 4.36		
20	R	89.52 ± 4.20	16.17	<0.05
	RC	84.20 ± 4.29		
25	R	92.46 ± 5.38	21.12	<0.05
	RC	82.46 ± 6.43		
30	R	86.56 ± 5.54	13.65	<0.05
	RC	80.16 ± 5.54		
40	R	85.48 ± 4.82	12.22	<0.05
	RC	81.56 ± 7.96		
50	R	86.38 ± 3.22	14.42	<0.05
	RC	80.48 ± 5.94		
60	R	82.46 ± 4.64	17.42	<0.05
	RC	75.68 ± 4.12		
75	R	78.18 ± 4.86	14.58	<0.05
	RC	72.44 ± 3.88		
90	R	76.44 ± 3.96	12.60	<0.05
	RC	70.46 ± 4.22		
105	R	75.92 ± 4.88	10.94	<0.05
	RC	70.58 ± 3.94		
120	R	74.16 ± 4.64	7.90	<0.05
	RC	70.12 ± 4.18		
150	R	70.24 ± 5.68	1.86	>0.05
	RC	69.90 ± 3.98		
180	R	70.42 ± 3.12	0.84	>0.05
	RC	70.16 ± 4.08		

The incidence of slight maternal tachycardia was comparable in both the groups up to 20 minutes, which can be explained on the basis of sympathetic block in the lower extremities and decreased venous return. But after 20 minutes of anesthetic dose, maternal heart rate kept on increasing in the R group but got stable in RC group and started decreasing thereafter which was statistically significant (*P* < 0.05). This can be possibly due to the effect of clonidine. The difference in the heart rate remained significant till regression of anesthetic effect in both the groups until 2 hours. Thereafter, the heart rate remained stable with hardly any fluctuation in either of the groups which is statistically nonsignificant (*P* > 0.05). Only two patients in R group and four patients in RC group had incidence of bradycardia with heart rate of <55 beats/minute and atropine had to be given in bolus doses of 0.3 mg [Table 4].

**Table 4:** Comparison of mean SBP at different time intervals in groups R and RC

Time interval (minutes)	Groups	Mean ± SD	SBP (Systolic blood pressure)	
			*t*	*P*
Baseline (1 minute)	R	124.38 ± 8.32	—	>0.05
	RC	122.42 ± 7.86		
5	R	122.46 ± 7.84	5.40	<0.05
	RC	119.26 ± 6.84		
10	R	121.54 ± 7.36	8.17	<0.05
	RC	114.36 ± 6.64		
15	R	117.22 ± 6.82	9.62	<0.05
	RC	110.18 ± 6.38		
20	R	116.18 ± 5.44	12.16	<0.05
	RC	106.30 ± 4.28		
25	R	112.96 ± 6.12	11.14	<0.05
	RC	108.26 ± 6.48		
30	R	111.90 ± 8.24	3.55	<0.05
	RC	109.26 ± 7.54		
40	R	112.42 ± 6.22	1.38	>0.05
	RC	111.56 ± 6.46		
50	R	111.28 ± 5.82	1.26	>0.05
	RC	110.98 ± 5.14		
60	R	113.26 ± 4.54	1.42	>0.05
	RC	112.88 ± 4.32		
75	R	115.20 ± 3.66	1.18	>0.05
	RC	114.14 ± 3.64		
90	R	114.29 ± 3.14	1.20	>0.05
	RC	113.24 ± 3.28		
105	R	116.16 ± 3.54	0.98	>0.05
	RC	115.98 ± 3.14		
120	R	118.14 ± 3.69	0.92	>0.05
	RC	117.92 ± 3.17		
150	R	117.64 ± 3.68	0.84	>0.05
	RC	118.10 ± 3.14		
180	R	118.22 ± 3.18	0.78	>0.05
	RC	117.96 ± 3.28		

The systolic blood pressure showed statistically significant difference (*P* < 0.05) in both the groups which coincided with the increasing sensory blockade in both the groups. This can be possibly due to the hypotensive action of clonidine in the RC group. But once the sensory and motor blocks were completely established, there was no significant difference of systolic BP in either of the groups (*P* > 0.05) [Table 5].

**Table 5:** Comparison of mean DBP at different time intervals in groups R and RC

Time interval (minutes)	Groups	Mean ± SD	Diastolic Blood pressure (DBP)	
			*t*	*P*
Baseline (1 minute)	R	82.24 ± 4.02	—	>0.05
	RC	81.88 ± 3.86		
5	R	80.16 ± 3.96	4.50	<0.05
	RC	78.34 ± 3.72		
10	R	80.45 ± 3.44	11.22	<0.05
	RC	75.43 ± 3.86		
15	R	77.86 ± 4.22	13.77	<0.05
	RC	71.62 ± 4.86		
20	R	76.56 ± 4.84	15.26	<0.05
	RC	69.28 ± 4.78		
25	R	73.28 ± 5.22	13.74	<0.05
	RC	68.76 ± 4.84		
30	R	70.58 ± 5.38	8.47	<0.05
	RC	67.72 ± 4.19		
40	R	70.39 ± 4.96	4.78	<0.05
	RC	68.10 ± 3.38		
50	R	71.65 ± 4.92	3.94	<0.05
	RC	69.29 ± 3.58		
60	R	71.34 ± 3.96	4.82	<0.05
	RC	69.72 ± 3.46		
75	R	73.35 ± 3.56	6.48	<0.05
	RC	70.71 ± 3.18		
90	R	76.65 ± 3.98	7.68	<0.05
	RC	72.76 ± 3.56		
105	R	77.35 ± 3.11	8.45	<0.05
	RC	73.68 ± 3.48		
120	R	76.34 ± 3.86	7.82	<0.05
	RC	72.68 ± 3.72		
150	R	77.46 ± 3.45	1.48	>0.05
	RC	76.88 ± 3.90		
180	R	78.76 ± 3.54	0.98	>0.05
	RC	77.98 ± 3.58		

The incidence of diastolic hypotension was more in RC group as compared to R group after the initiation of sympathetic block. This can again be explained on the basis of hypotensive action of clonidine causing a prolonged and significant diastolic hypotension in our patients (*P* < 0.05). The action of clonidine on diastolic component remained for almost 2 hours in our RC study group. Thus, requirement of mephenteramine was much higher in RC group as compared to R group. All patients were treated with incremental doses of mephenteramine 3 mg bolus doses but the total dose did not cross 18 mg [Table 6].

**Table 6:** Comparison of side effects in groups R and RC

Side effects	R	RC	*P*
Nausea/vomiting	4	6	>0.05
Sedation	0	2	
Shivering	3	1	>0.05
Respiratory depression	0	0	
Headache	1	2	>0.05
Dry mouth	2	9	<0.05

The two groups were almost comparable as far as the side effects are concerned. There was no significant difference in both the groups with regard to nausea, vomiting, sedation, shivering, respiratory depression or headache (*P* > 0.05). Nine patients complained of dry mouth as compared to none in the R group which was statistically significant (*P* < 0.05). This can be attributed to the side effects of clonidine [Table 7].

**Table 7:** Comparison of Apgar scores in neonates of R and RC groups

Delivery parameters	Ropivacaine (*n* = 24)	Ropivacaine + clonidine (*n* = 27)
Weight (kg)	3.046 ± 0.546	3.104 ± 0.468
Period of gestation (days)	274.58 ± 8.64	275.15 ± 9.66
First min Apgar score (mean)	8	8
Fifth min Apgar score (mean)	10	10
Tenth min Apgar score	10	10

Neonatal Apgar scores were assessed at 1, 5, 10 minute intervals. There were three patients in RC group who had Apgar score of less than 7 at 1-minute interval as compared to one neonate in the R group, which was statistically not significant. But one of the neonates in RC group had breech presentation with cord around the neck. At 5-minute intervals, all the neonates in RC and R groups had Apgar score of more than 9 which was statistically nonsignificant. At 10-minute intervals, the Apgar score in both the groups were comparable and were approximately 10 and showed no statistical significant difference. No neonate was admitted to the neonatal intensive care unit for intensive monitoring. Pediatricians reported a normal clinical status within the first 24 hours in all three neonates born to mothers receiving clonidine and one neonate whose mother did not receive clonidine in whom abnormal Apgar scores (<7) were recorded at 1-minute interval [Table 8].

**Table 8:** Comparison of peroperative and postoperative block characteristics in R and RC groups

Block characteristics	R (*n* = 24)	RC (*n* = 27)	*P*
Time to segmental regression (minutes)	88.32 ± 16.52	102.80 ± 18.38	<0.05
Time to segmental regression to S2	112.56 ± 11.34	128.16 ± 13.48	<0.05
Duration of anesthesia (minutes)	131.40 ± 24.40	173.50 ± 32.44	<0.05
Time for first top-up (minutes)	117.49 ± 22.34	138.46 ± 25.42	<0.05
Time interval between postoperative supplementary analgesic doses (hours)	4.56 ± 1.28	6.23 ± 1.46	<0.05
Total dose of ropivacaine postoperatively (mg)	230.76 ± 26.28	150.34 ± 21.46	<0.05

Postoperatively, the two-segment dermatome regression and onset of pain was much earlier in the R group as compared to RC group. The requirement of ropivacaine, 8 mL of 0.25%, for top-up doses was significantly reduced in the RC group as compared to the R group. Clonidine was added at a dose of 50 µg to 8 mL solution of 0.25% ropivacaine in the top-up bolus doses of RC group, whereas the R group received only 8 mL of 0.25% ropivacaine. The time interval of top-up doses varied significantly with increased time interval between successive doses in the RC group (*P* < 0.05). The total dose requirement of ropivacaine significantly increased in RC group.

## DISCUSSION

There are numerous studies on the use of epidural ropivacaine for inducing painless labor and deliveries.[9] The addition of various adjuvants to epidural ropivacaine for achievement of labor analgesia with subsequently lesser dose requirement of the ropivacaine has been studied to some extent.[2] Although intrathecal clonidine as an adjuvant to ropivacaine has been studied extensively, till date there has been hardly any study which has detailed data on the epidural use of ropivacaine and clonidine for cesarean section. Though ropivacaine is slightly less potent as compared to bupivacaine, its pharmacological profile is almost comparable to the latter. Various studies and literary evidence have concluded that cardiotoxicity of ropivacaine is far less than that of bupivacaine.[10,11] Dose of ropivacaine is much higher as compared to bupivacaine when used epidurally. To achieve sufficient analgesia with ropivacaine for cesarean section operation or major abdominal surgery, the dose of ropivacaine should be higher than bupivacaine which may lead to adverse local anesthetic side effects especially in obstetric patients.[12]

The present study was undertaken to compare the epidural ropivacaine with a combination of epidural ropivacaine–clonidine for elective cesarean sections with major emphasis on onset, intensity and duration of block, maternal cardiorespiratory parameters, postoperative analgesia and Apgar score. We chose only epidural route and not combined spinal epidural because there have been no studies comparing these drugs through epidural route solely. Secondly, epidural route does provide a better control on maternal hemodynamic parameters as compared to intrathecal drugs. Thirdly, we also wanted to provide our patients a postoperative pain-free stay in our hospital and to avoid systemic analgesics. Finally, it was an indirect effort to educate these patients about the beneficial effects of regional epidural anesthesia thereby removing their false notions associated with the pain and other misinterpretations of regional anesthesia, which are so much prevalent in our society.

The high cephalic spread of ropivacaine analgesia may be significant but still its quality may not correlate with the level of sensory analgesia.[13] The addition of clonidine as an adjuvant allows a lesser dose of ropivacaine when used intrathecally for cesarean section.[14] Clonidine augments the action of local anesthetics in regional blockades by interrupting the neural transmission of painful stimuli in Aδ and C fibres as well as augments the blockade of local anesthetic agents by increasing the conductance of K^+^ ions in nerve fibres. It also exerts a vasoconstricting effect on smooth muscles, which results in a decreased absorption of the local anesthetic drug and eventually prolongs the duration of analgesia.[15,16] Keeping all these pharmacological interactions in mind, we have tried to use clonidine as an adjuvant to ropivacaine not just covering the operative period, but the postoperative period as well. Neuraxial opioids are associated with quite a few side effects in parturients; so, clonidine is being extensively evaluated as an alternative option as far as opioid-related side effects such as respiratory depression, nausea and pruritis are concerned.[17–19]

The attempts done earlier for dose-determination concluded that 75 µg of clonidine is the optimal epidural dose when added to bupivacaine for labor analgesia as smaller doses were not serving the purpose of adequate analgesia, whereas larger doses were associated with side effects like maternal bradycardia, hypotension, sedation and deleterious side effects on fetal heart rate.[20] Therefore, we administered single clonidine dose of 75 µg for operative purpose, whereas top-up doses of 50 µg clonidine were administered with 0.25% ropivacaine for the postoperative pain relief. This was done to have minimal side effects on fetal cardiac tissue during surgical procedure as cumulative doses administered before delivery can have fetal heart rate abnormalities.[21,22]

In this study RC group had shorter onset time of analgesia as compared to R group which is almost consistent with other studies.[14,23] In the previous studies involving the combination of bupivacaine with clonidine, there was hardly any finding of shorter onset time of analgesia which encouraged us to use the combination of ropivacaine and clonidine.[20,24]

The addition of clonidine is better than the opiods when side effects on the maternal physiology as well as the fetal Apgar scores are considered.[25] Few parturients did require mephenteramine but the systolic/diastolic pressure in these patients was never allowed to fall significantly thus rendering the uterine placental perfusion in jeopardy. Sedation was observed in only two patients of RC group which can be again explained on the basis of lower dose of clonidine. There has been no literary evidence that has shown the adverse effects on the neonates when clonidine is used as an adjuvant with local anesthetic either by epidural or intrathecal routes. In previous labor studies, clonidine added to local anesthetic in doses between 15 and 150 µg was reported to cause mild to moderate hypotension without any effect on neonatal outcomes.[7,25] These facts helped us further to explore the synergistic interaction of these two drug combinations as well as dose reduction of local anesthetic thereby limiting the low risk of cardiotoxicity of ropivacaine even still further. There are no statistical data available in literature indicating the impact of maternal cardiovascular effects which influences the neonatal outcome. Since this study was designed mainly to know the impact of combination on quality and duration of anesthesia, the effect on fetal parameters was not stressed upon in detail except the Apgar score. This was mainly done on the basis of proven statistical records of limited effect of clonidine on fetal well-being with the use of 75 µg epidural dose.[22,25] Ropivacaine does neither have any detrimental effect on uteroplacental and fetal circulation measured by Doppler recordings nor does it have any detrimental effect on Apgar scores or acid-base values in the umbilical blood flow after delivery.[26]

Previous studies conducted for labor analgesia have used 75 µg of clonidine with a lower concentration of ropivacaine but since our patients had to undergo surgical procedure we used 75 µg of clonidine but with much higher concentration of ropivacaine for profound anesthetic effect. Our study confirms the fact that the combination of 75 µg of clonidine provided shorter onset and cephalic spread but longer sensory analgesia when compared to a similar dose of plain ropivacaine. The motor block was also complete almost 25–30 minutes after the administration of drugs in both the groups but much earlier in the RC group. The results of our study have further helped us to conclude that epidural ropivacaine combined with epidural clonidine causes profound motor block with rapid onset and prolongs the duration of analgesia as compared to plain ropivacaine. Epidural anesthesia for elective cesarean section has got an advantage in that analgesia can be prolonged in the postoperative period and further helps in smooth shifting of patients to recovery beds and better maternal comfort with top-up doses of local anesthetics.

Doses and routes of administration of clonidine are mainly responsible for majority of its side effects namely hypotension, bradycardia, sedation, drowsiness, etc[3,4] Larger doses like 150 µg of clonidine when used in spinal anesthesia have notably caused hypotension, sedation and dry mouth but no delayed side effects especially related to hemodynamic parameters in women undergoing cesarean section operations.[18] The interaction of clonidine with central α-2 receptors causes sedation while augmentation of parasympathetic system and inhibition of sympathetic outflow activity are mainly responsible for centrally mediated hemodynamic effects. These effects become more pronounced with larger doses of clonidine.[4,17] Few patients developed moderate hypotension in both the groups, which was treated with the injection mephenteramine up to maximum doses of 18 mg. The requirement of mephenteramine was slightly higher in the clonidine group but it was not clinically significant. Hemodynamic side effects like hypotension and bradycardia neither had any major impact nor any sequel on the peroperative or postoperative course of mother or on the neonates. No neonate showed any sign of sedation after delivery and not a single infant required admission to nursery for intensive care. We did the follow-up of infants for 24 hours after the surgery and did not find any untoward incident which also corroborated the evidence that 75 µg of epidural clonidine does not cause any side effects in the neonates. Postoperatively, maternal comfort was to the utmost as far as analgesia was concerned. The patients of RC group required lesser doses of local anesthetic top-up doses as compared to that required by R group patients. The frequency of top-up doses increased, duration of analgesic period decreased while total dose consumption of ropivacaine increased in the R group as compared to RC group. Maternal comfort, assessment of quality of anesthesia and prolonged painless period were the highlights of postoperative period which was slightly better perceived by the RC group.

## CONCLUSIONS

Epidural 0.75% of isobaric ropivacaine provides efficient and safe anesthesia for cesarean section delivery. The addition of 75 µg clonidine to isobaric ropivacaine results in longer complete and effective analgesia with similar block properties and helped to reduce the effective dose of ropivacaine and improved the intraoperative surgical conditions when compared with plain ropivacaine for cesarean delivery. Clonidine combination with ropivacaine did not affect maternal and neonatal outcome. We recommend the regular use of epidural ropivacaine and clonidine for elective cesarean sections as far as safety of drugs, better peroperative conditions, unaffected Apgar scores, postoperative maternal comfort and uneventful neonatal outcome are concerned.
